# Rapid Spectroscopic Liquid Biopsy for the Universal Detection of Brain Tumours

**DOI:** 10.3390/cancers13153851

**Published:** 2021-07-30

**Authors:** Ashton G. Theakstone, Paul M. Brennan, Michael D. Jenkinson, Samantha J. Mills, Khaja Syed, Christopher Rinaldi, Yun Xu, Royston Goodacre, Holly J. Butler, David S. Palmer, Benjamin R. Smith, Matthew J. Baker

**Affiliations:** 1 Technology and Innovation Centre, Department of Pure and Applied Chemistry, University of Strathclyde, Glasgow G1 1RD, UK; christopher.rinaldi@strath.ac.uk; 2 Translational Neurosurgery, Centre for Clinical Brain Sciences, University of Edinburgh, Edinburgh EH16 4SB, UK; paul.brennan@ed.ac.uk; 3 The Walton Centre NHS Foundation Trust, Lower Lane, Liverpool L9 7LJ, UK; Michael.Jenkinson@liverpool.ac.uk (M.D.J.); Samantha.Mills@thewaltoncentre.nhs.uk (S.J.M.); khaja.syed@thewaltoncentre.nhs.uk (K.S.); 4 Department of Pharmacology & Therapeutics, Institute of System, Molecular and Integrative Biology, University of Liverpool, Liverpool L69 7ZB, UK; 5 Department of Biochemistry and Systems Biology, Institute of Systems, Molecular and Integrative Biology, University of Liverpool, Liverpool L69 7ZB, UK; Yun.Xu@liverpool.ac.uk (Y.X.); roy.goodacre@liverpool.ac.uk (R.G.); 6 Dxcover Limited, 204 George Street, Glasgow G1 1XW, UK; holly.butler@dxcover.com (H.J.B.); david.palmer@strath.ac.uk (D.S.P.); benjamin.smith@dxcover.com (B.R.S.); 7 Department of Pure and Applied Chemistry, University of Strathclyde, Thomas Graham Building, Glasgow G1 1XL, UK

**Keywords:** vibrational spectroscopy, glioma, chemometrics, clinical translation, early detection

## Abstract

**Simple Summary:**

Due to the non-specific symptoms of brain cancer (e.g., headaches or memory changes), gliomas will often remain undetected until they are larger or at a higher grade, reducing the patient’s likelihood of a good clinical outcome. Earlier detection and diagnosis of brain tumours is vital to improve patient outcomes, leading to safer surgeries and earlier treatments. A liquid biopsy for brain tumour would prove revolutionary however in order to detect disease earlier the liquid biopsy needs to be able to detect smaller tumours; and current liquid biopsies perform worse when detecting smaller or earlier stage tumours. Here, for the first time, we confirm the applicability of a validated spectroscopic liquid biopsy approach to detect both small and low-grade gliomas proving that the spectroscopic liquid biopsy approach is insensitive to tumour volume unlike other liquid biopsies.

**Abstract:**

Background: To support the early detection and diagnosis of brain tumours we have developed a rapid, cost-effective and easy to use spectroscopic liquid biopsy based on the absorbance of infrared radiation. We have previously reported highly sensitive results of our approach which can discriminate patients with a recent brain tumour diagnosis and asymptomatic controls. Other liquid biopsy approaches (e.g., based on tumour genetic material) report a lower classification accuracy for early-stage tumours. In this manuscript we present an investigation into the link between brain tumour volume and liquid biopsy test performance. Methods: In a cohort of 177 patients (90 patients with high-grade glioma (glioblastoma (GBM) or anaplastic astrocytoma), or low-grade glioma (astrocytoma, oligoastrocytoma and oligodendroglioma)) tumour volumes were calculated from magnetic resonance imaging (MRI) investigations and patients were split into two groups depending on MRI parameters (T1 with contrast enhancement or T2/FLAIR (fluid-attenuated inversion recovery)). Using attenuated total reflection (ATR)-Fourier transform infrared (FTIR) spectroscopy coupled with supervised learning methods and machine learning algorithms, 90 tumour patients were stratified against 87 control patients who displayed no symptomatic indications of cancer, and were classified as either glioma or non-glioma. Results: Sensitivities, specificities and balanced accuracies were all greater than 88%, the area under the curve (AUC) was 0.98, and cancer patients with tumour volumes as small as 0.2 cm^3^ were correctly identified. Conclusions: Our spectroscopic liquid biopsy approach can identify gliomas that are both small and low-grade showing great promise for deployment of this technique for early detection and diagnosis.

## 1. Introduction

Earlier diagnosis of patients with brain cancer can greatly improve patient outcomes. When a tumour is smaller, surgery is safer and can be more extensive with less morbidity and damage to the patient. Extent of surgical resection correlates with clinical outcome [1] and earlier surgery may improve survival. Whilst no therapy for gliomas is currently curative, new novel therapies are more likely to be effective in patients with smaller tumours.

However, brain cancer diagnosis is difficult. Common brain cancer symptoms such as headaches or memory change are non-specific and more likely to be associated with a non-cancer diagnosis [2]. Patients visit their primary care doctor on average three or more times before diagnosis and two thirds of patients are diagnosed in the Emergency Department when their symptoms have deteriorated [3]. More rapid access to diagnostic brain imaging from primary care could help [4]. However, most patients referred with suspected cancer for brain imaging do not have a cancer. With open access brain imaging for patients with suspected brain cancer only 1.8% of scans identify a brain tumour [5]. New strategies are thus needed that can be deployed in primary care for both symptomatic and asymptomatic patients with a low suspicion of cancer; in order to identify which patients could be prioritised for urgent brain imaging.

Assessment of tumours is initially completed through MRI, typically acquired either by T1- or T2-weighted protocols which differ by the timing of the radiofrequency pulse sequences and relaxation times. T1-weighted images brighten the fatty tissues within the body with cerebrospinal fluid (CSF) appearing black as it contains no fat. T2-weighted images brightens both the fatty tissues as well as water so any water-based tissue can be distinguished from fatty tissue by comparing to the T1 image [6,7]. Tumours have variable shape and size requiring a segmented approach when calculating volume which can be hindered depending on location and imaging parameters [8,9]. Accuracy is most likely to be reduced with the T2 and fluid-attenuated inversion recovery (FLAIR) volumes.

In a recent prospective clinical trial, our ATR-FTIR spectroscopy-based liquid biopsy test correctly determined which of 385 patients with suspected brain cancer referred from primary care for brain imaging had cancer, with 81.0% sensitivity and 80.0% specificity. The clinical feasibility of this technique was explored for the triage of symptomatic patients whose symptoms might be indicative of a brain tumour. This rapid (15 min is typical per patient sample), low-cost test can integrate with clinical assessment in primary care to help identify which patients to prioritise for diagnostic imaging; to help achieve earlier cancer detection and diagnosis.

Health economic evaluation in terms of cost-effectiveness and cost-consequence analysis has been completed for both retrospective patient data as well as the recent prospective clinical trial [10,11]. Evaluations were completed when introducing the spectroscopic liquid biopsy as a triage to brain imaging in both primary and secondary care scenarios. The results from the retrospective study indicated that this technology would be more effective than current tests in both scenarios and cost saving for health services within primary care [10]. From the more recent clinical trial it was evaluated that this technology is cost-effective for both primary and secondary care settings [11].

Infrared spectroscopy is a phenotypic method, which quantifies the absorption of mid-infrared light (4000–450 cm^−1^) by molecules such as lipopolysaccharides, lipids, carbohydrates, nucleic acids, and proteins, resulting in a specific FTIR spectrum that reflects the overall composition of the sample [12]. Commercially available silicon internal reflection elements (SIREs), such as the ones used in this study, offer multiple sampling points, repeat analysis if required, and are relatively low cost suited for routine diagnostic use.

Vibrational spectroscopy techniques such as ATR-FTIR require only small amounts of material (µL range), is non-destructive, reproduceable, and therefore continues to be utilised for biological, chemical and clinical studies [13,14,15]. Specific molecular characteristics, such as functional groups or chemical bonds provide information on molecular arrangement and are assigned as FTIR peaks, with any changes that are related to disease can be measured as characteristic to either diseased or non-diseased patients through the use of machine learning algorithms [15,16,17].

Liquid biopsies are an emerging field within early detection and diagnosis of diseases. The majority of reports focus on a genomic based approach where genetic material such as circulating DNA is targeted [18,19]. In both cancer patients and healthy individuals, the circulating DNA levels vary by 1 to 2 orders of magnitude [20,21], with normal individuals having circulating free DNA (cfDNA) within the range of 1–10 ng/mL [22,23]. A study by Fiala et al. outlined that when the fraction of DNA is below 1 circulating tumour DNA (ctDNA) to 10,000 total circulating DNA then 10 mL of patient blood (4 mL of plasma) will not contain a single genome ctDNA for sequencing and diagnosis of cancer is impossible [24]. This poses a significant issue with the genomic based approaches as large volumes of patient blood would be required to detect the amount of cancerous genetic material present, especially at an early stage where the genetic material available is incredibly low [25].

Early cancer detection and diagnosis requires that a tumour can be identified when it is as small as possible, and when a patient will often have few or no symptoms. Here, we have examined the performance of a rapid spectroscopy-based liquid biopsy test in relation to tumour volume in a cohort of patients with a new brain cancer diagnosis. The spectral data coupled with machine learning algorithms will differentiate between these cancer patients and asymptomatic controls in order to identify the tumour volume range that is detectable using this developed technique for stratification.

## 2. Materials and Methods

### 2.1. Patient Selection

A total of 177 serum samples were obtained from the Walton Centre NHS Trust (Liverpool, UK) and the Royal Preston Hospital (Preston, UK) with patient consent, under Ethics approval code (Walton Research Bank BTNW/WRTB 13_01/BTNW Application #1108). Of the 177 patients there were 87 asymptomatic controls (no brain cancer), 47 patients with high-grade (grade IV) glioblastoma (GBM), 13 with grade III anaplastic astrocytoma (AA), and 30 with low-grade (grade II) gliomas (LGG) [including 12 astrocytoma (A), 3 oligoastrocytoma (OA) and 15 oligodendroglioma (OD)]. Tumour volumes were measured by a consultant neuroradiologist using the assessment tool on Carestream PACS from T1-weighted with contrast-enhancement, T2-weighted or FLAIR MRI. Both T2-weighted and FLAIR images were collected without the use of contrast-enhancement. Patients were matched for gender, age was between 30 to 82 years old, and tumour sizes ranged from 0.2 cm^3^ to 226.2 cm^3^ as outlined in Supplementary material Table S1.

The cancer patients were split into two groups, T1-weighted with contrast-enhancement and T2-weighted/FLAIR. The T1 cohort consisted solely of GBM tumours as a more accurate measurement of high-grade gliomas requires the use of a contrasting agent [26]. The T2/FLAIR group comprised of both grade III AA and all the LGG. The grade III AA were included with the LGG as the radiological appearances of AA varies with contrast enhancement and it was deemed more accurate to use the measurements of these tumours from their T2/FLAIR images.

### 2.2. Spectroscopic and Data Analysis

Patient serum (3 µL) was deposited onto a SIRE optical sample slide and spread using a pipette tip across the whole well to provide a uniform deposition. The slides were then placed into a drying unit incubator for 1 h ensuring serum was dried before spectroscopic data collection. All serum spectra were collected on a Perkin Elmer Spectrum 2 FTIR spectrometer (Perkin Elmer, London, UK), utilising a Specac Quest ATR accessory unit with a specular reflectance puck (Specac Ltd., London, UK), allowing a Dxcover optical sample SIRE (Dxcover Ltd., Glasgow, UK) to be placed directly on top of the aperture. Nine spectra per patient were collected within the range of 4000–450 cm^−1^, at a resolution of 4 cm^−1^, with 1 cm^−1^ data spacing and 16 co-added scans; resulting in a total of 1593 spectra acquired. The typical time for spectral collection was 15 min per patient sample slide (9 repeats and background).

The spectroscopic data analysis was completed either using R Statistical Computing Environment or MATLAB R2020a software with the PRFFECT toolbox [27], the principal component analysis (PCA) code, receiver operator characteristic (ROC) curve code, and PLS-DA bootstrapping code used for resampling and permutation analysis is freely available at https://github.com/biospec (accessed on 29 January 2021). The PCA and ROC curve codes were written in-house. Data pre-processing was a trial-and-error iterative approach utilising the PRFFECT toolbox and was completed before each PCA or classification. Numerous pre-processing options were explored in order to reduce computational burden and improve classification algorithms by removing unwanted noise and/or artefacts. Some of the common pre-processing techniques included data binning, normalisations, and baseline corrections [27,28]. Binning averages adjacent data points in order improve the signal-to-noise ratio, lowers the dimensionality of the dataset and reduces computational burden [27,28,29]. Normalisation includes techniques such as min-max scaling (between 0 and 1), which scales the entire spectrum to a range between a minimum absorbance of 0 and a maximum absorbance of 1; vector normalisation which ensures all spectra have a vector length of 1 resulting in mean centering and scaling; as well as normalisation to the Amide I or Amide II band, where the spectra is scaled to the intensity of the absorbance in those particular wavenumber regions. (Amide I 1700–1600 cm^−1^ and Amide II 1600–1450 cm^−1^) [28,30]. Baseline corrections include Savitzky-Golay derivative filters (1st and 2nd), polynomial, rubberband and extended multiplicative signal correction (EMSC). Derivative filters improve spectral resolution with a straightforward mathematical transformation, polynomial baseline corrections are mostly used with Raman spectroscopy, rubberband baseline corrections fits a convex polygonal to the troughs of the spectrum in order to adjust the baseline while EMSC uses a reference spectrum to scale each datapoint [27,31].

The optimum pre-processing techniques for this data involved a min-max normalisation, a binning factor of 8, cutting to the spectral region of 1800–1000 cm^−1^, and an EMSC which used the average spectrum of 10 background measurements of the SIRE as a reference.

Exploratory analysis was completed using PCA, which involves an orthogonal linear transformation to determine any natural separation between the two classes (glioma or non-glioma). The resulting scores plots allow for any variance to be displayed as principal components (PC) with the first PC accounting for greatest variance. The corresponding loading plots are useful in identifying the specific wavenumber regions that are responsible for any variability between the two classes [30].

There are multiple supervised learning methods and machine learning algorithms that are useful for disease diagnostics or classifications including random forest (RF), partial least squares-discriminant analysis (PLS-DA) and support vector machine (SVM) that were applied here. Each technique requires splitting the data into training and test sets where the training set is used to identify biosignatures in a calibration phase and the model generated subsequently used for predictions to be made on the test set [15,30,32,33]. RF uses a Classification and Regression Trees (CART) algorithm to build an ensemble of decision trees as independent base models which predicts as a majority vote within the forest [34,35]. PLS-DA uses PLS regression with a dummy response matrix of 0 and 1 s to represent different classes [36]. SVM separates two classes of data by finding an optimal boundary defined by a subset of data points of the two different classes which are nearest to each other, known as support vectors [30]. As with any technique, each model has its own advantages and disadvantages which are highlighted in a recent review and adapted from Gromski et al. [37,38]. Sensitivity, specificity, and balanced accuracy all contribute to the performance of the algorithms with sensitivity referring to the predictive ability of true positives, specificity referring to the predictive ability of true negatives, and balanced accuracy referring to the overall performance of the model [30]. Definitions and mathematical equations of sensitivity, specificity and balanced accuracy is included within the Supplementary materials. A ROC curve can also be generated in order to measure the performance capabilities with the area under the curve (AUC) representing a degree of separability between the groups [39].

Sampling techniques such as up-sampling, down-sampling and synthetic minority over-sampling technique (SMOTE) are also useful for classification analysis depending on the number of patients in each class and whether or not there is an imbalance [30]. Up-sampling increases the size of minority group by allowing the same sample to be sampled multiple times, down-sampling reduces the size of the majority group by sampling less data from the majority, where SMOTE creates a more balanced dataset by generating sufficient synthetic samples for minority group by artificially mixing the data [30]. Due to imbalance in the case–control groups a SMOTE sampling technique was used for all classification analysis presented here.

Each classification completed using the PRFFECT toolbox had 51 reiterations to minimise standard error and to ensure a robust diagnostic model was used. The data were randomly split by patient ID at a 70/30 ratio between the training and test sets, keeping all patient spectral repeats together. The 51 reiterations shuffled the 70/30 split each time so that every patient within the whole dataset was predicted at least once. For both the T1 and T2/FLAIR volumes, the classification was glioma versus non-glioma to gain the predictive ability of the model in order to identify which patients were, if any, misclassified, and what size tumour that corresponds with.

A total number of 1000 permutation tests were completed where the labels were randomised in order to assess the statistical significance of the classification model to obtain an empirical *p*-value. This was done through PLS-DA classification modelling with SMOTE sampling and 1000 bootstrapping validations. With-in each bootstrapping validation, a PLS-DA model was trained by using known labels (observed model) while another PLS-DA model was training by using randomly permuted labels (null model). The predictive accuracies of these two sets of models formed two distributions: an observed distribution and a null distribution. A good separation between these two distributions would suggest that the separation found by the PLS-DA models were statistically significant otherwise it would suggest that the separation was happened by chance. An empirical *p*-value was also derived by calculating the percentages of cases when null models had obtained better accuracy than observed models [40].

## 3. Results

For the T1 gadolinium enhanced MRI tumour volumes (diagnostic investigation for high-grade glioma), exploratory PCA was conducted in order to explain any variance within the data between the two groups of interest; enhanced tumour volume and control patients. A scree plot was used to illustrate the percentage of data variance present in each principal component (PC) with 62.7% of variance within the first PC, 12.8% within the second and 5.7% within the third as shown within the Supplementary materials (Figure S1). The PCA plot illustrated a separation between the cancer and control patients along the second PC as displayed within Supplementary materials Figure S2.

From this a contribution plot (Supplementary materials Figure S3) was obtained which displays the particular wavenumbers within the spectral data that are responsible for the separation between the two groups in the second dimension. These wavenumbers can be assigned to particular biochemical functional groups and are important when distinguishing between glioma and non-glioma patient spectra. The wavenumbers between 1100–1000 cm^−1^ correspond to the stretching vibrational modes of C-O (carbohydrates) and PO^2−^ (nucleic acids). Wavenumbers between 1600–1450 cm^−1^ represent the vibrational modes of the Amide II of proteins; N-H (bending), C-N (stretching), C-O (bending) and C-C (stretching), and 1700–1600 cm^−1^ can be assigned to the C-O (stretching), C-N (stretching) and N-H (bending) of the Amide I of proteins. The machine learning techniques are required to identify any spectral variations between the two groups with an example spectra of the glioma cohort presented within the Supplementary materials (Figure S4).

Following this exploratory technique between the cancer patients versus controls, three different classification models (PLS-DA, SVM, and RF) were explored in order to stratify between the two classes. Each classification was reiterated 51 times which was deemed an acceptable number of repeats to minimise both sensitivity and specificity error whilst also minimising analysis time, as reported by Cameron et al. on similar vibrational spectroscopic data [30]. As displayed in Table 1 the PLS-DA model had the greatest predictive ability with a sensitivity of 98.5%, specificity of 95.1% and balanced accuracy of 96.8%. All classifications here gave sensitivities, specificities and balanced accuracies above 90% which is consistent with previous work introducing the use of similar spectroscopic data with machine learning algorithms for stratifying between cancer and non-cancer patients [30].

Similar to the T1 enhanced volume group, patients with T2 or FLAIR MRI tumour volumes (diagnostic investigation for low-grade glioma) were subjected to exploratory PCA as well as each classification (PLS-DA, SVM, and RF). A scree plot illustrated 66.4% of data variance between cancer versus control patients within the first PC, 9.3% for PC2 and 5.4% within the third PC; as displayed within the Supplementary materials (Figure S5). The PCA plot (Figure S6) resulted in a similar pattern to that of the T1 enhanced volume patients with a separation between the two classes along the second dimension. The contributions plot (Figure S7) also corresponds with the previous group outlining the wavenumbers that are important for the separation between the cancer and control patients.

The sensitivity, specificity and balanced accuracy for all classifications with T2/FLAIR volumes is displayed in Table 1, with PLS-DA once again performing the greatest out of the three algorithms. All classifications had balanced accuracies above 88%, with PLS-DA resulting in a sensitivity of 88.7%, specificity of 94.7% and balanced accuracy of 91.7%.

**Figure 1:**
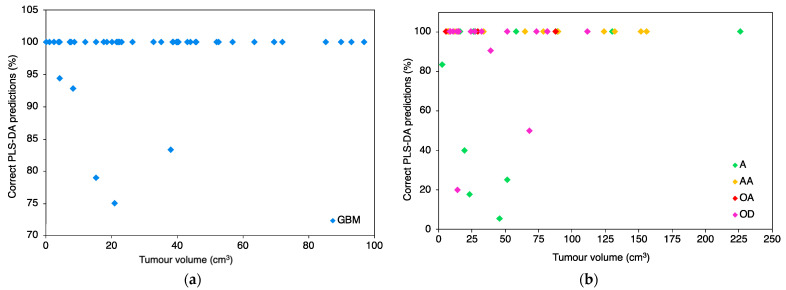
Percentage of correct cancer predictions for every patient over nine repeats and 51 iterations with the partial least squares-discriminant analysis (PLS-DA) classification model. T1 cohort (**a**) and T2/FLAIR cohort (**b**).

Every patient within the test set was classified as either glioma or non-glioma for each iteration; with the first iteration results displayed in Table S2 for the cancer patients. For each reiteration the test set contained different patients so that every patient within the whole dataset was predicted at least once. The average percentage of correct predictions for every cancer patient is displayed in Figure 1. The T1 cohort consisted solely of high-grade GBM tumours while the T2/FLAIR group contained predominately low-grade tumours [astrocytoma (A), oligoastrocytoma (OA) and oligodendroglioma (OD)] and higher-grade anaplastic astrocytoma (AA) where the diagnostic imaging did not show post gadolinium enhancement.

The first iteration for each classification model gave correct predictions for all of the cancer patients within the T1 cohort and majority within the T2/FLAIR. This is a promising result as it suggests this technique is capable of stratifying cancer patients with tumours as small as 0.2 cm^3^. Figure 1 considers the average percentage of predictions for all nine spectra of each cancer patient for all 51 reiterations. For the PLS-DA classification it is evident that majority of the patients were correctly identified as a cancer patient 100% of the time, over 51 reiterations and nine repeats. Within the T1 cohort, 5 out of the 47 cancer patients were mispredicted some of the time over the 51 reiterations however they were still correctly identified 75.0%, 78.9%, 83.3%, 92.9% and 94.4% of the time (Figure 1a). The T2/FLAIR group displayed greater variance with percentage of correct predictions (Figure 1b) over the nine patient repeats and 51 reiterations. Majority of the patients were correctly identified as a glioma patient 100% of the time however 8 out of the 43 were incorrect a number of times. The correct percentages of these 8 patients were 5.6%, 17.6%, 20.0%, 25.0%, 40.0%, 50.0%, 83.3% and 90.5% (Figure 1b).

**Figure 2:**
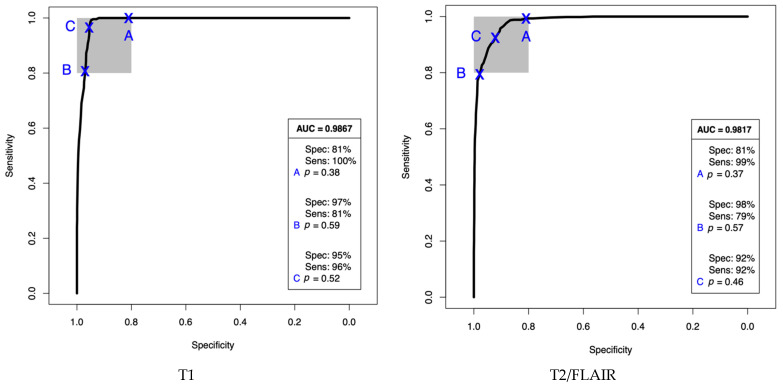
ROC curves with AUC for T1 and T2/FLAIR patient classification over nine repeats and 51 iterations with the partial least squares-discriminant analysis (PLS-DA) model.

A ROC curve was performed in order to display the diagnostic capability of the classification models with the area under the curve (AUC) calculated for both groups of patients (Figure 2). With a lower threshold (point A) we can calculate the effect on specificity with maximum sensitivity, and a higher threshold (point B) will favour specificity, lowering sensitivity. Point C refers to the optimal threshold for these binary classifications in order to produce both high sensitivity and specificities. The AUC for both the T1 and T2/FLAIR patient cohorts (0.9867 and 0.9817, respectively) represents an excellent measure of separability between the glioma and non-glioma groups.

**Figure 3:**
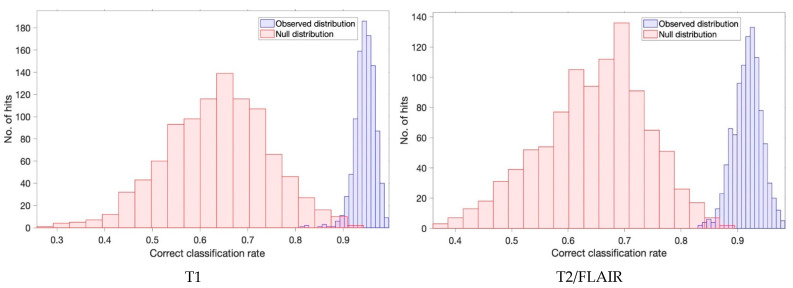
Null (red) and observed (blue) distribution classification rates for both the T1 and T2/FLAIR cohorts with a PLS-DA classification model after 1000 bootstraps.

Permutation tests were completed to assess the statistical significance of these classification findings with PLS-DA coupled with 1000 bootstrapping validations. The correct classification rate (CCR) for both T1 and T2/FLAIR was reported close to 1 (0.95 and 0.92, respectively), which illustrates excellent distinction and separation between the null and observed distributions. The null hypothesis suggests that the separation between the two groups (glioma and non-glioma) found by the PLS-DA model was happened by chance, therefore we want to reject the null hypothesis to support that the classification results were genuine. Figure 3 displays the null and observed distribution histograms for both T1 and T2/FLAIR, illustrating the support of the machine learning classification from the null hypothesis.

From this analysis a *p*-value was calculated in order to confirm statistical significance with both tumour volume patient cohorts < 0.01 (T1 = 0.004 and T2/FLAIR = 0.001), which indicates strong evidence against the null hypothesis. This suggests that there is a 0.4% and 0.1% chance the null hypothesis is correct, respectively.

**Figure 4:**
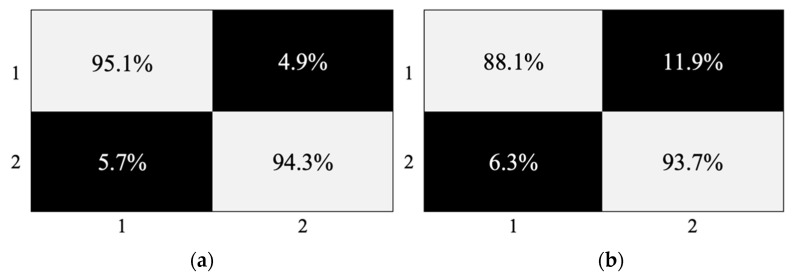
Confusion matrix illustrating the percentage of TP, FP, TN and FN for both the T1 (**a**) and T2/FLAIR (**b**) cohorts with a PLS-DA classification model after 1000 bootstraps.

A confusion matrix from this machine learning classification (Figure 4) displays the true positives (TP—95.1% and 88.1%), false positives (FP—4.9% and 11.9%), true negatives (TN—94.3% and 93.7%) and false negatives (FN—5.7% and 6.3%), between the two classes (Class 1 = glioma and Class 2 = non-glioma). These percentages correspond well with the sensitivity and specificity values reported above and once again confirm the use of these classification models for glioma versus non-glioma predictions.

## 4. Discussion

Liquid biopsies are emerging as a powerful new tool for tumour detection, as they are minimally invasive and more easily deployed at scale. Many liquid biopsies involve genomic approaches (e.g., tumour cell free (cf) DNA) aiming to detect cancerous genetic material. For early cancer detection there are several limitations with this approach. Early-stage tumours have very low mutation information systemically which is beyond current limits of detection; it can be less than one mutation per millilitre of plasma [25]. In CNS tumours genetic material may not pass through the blood–brain barrier [25]. Another limitation is that mutations in cfDNA can be found in the healthy non-cancer population [24] and that many mutations are characteristic of multiple cancer types, so localisation is not possible.

Rather than detecting specific genetic material, our strategy is to focus on analysing the global molecular phenotype of a blood sample. Our results demonstrate that patients produce systemic evidence of their CNS tumour that is readily detectable via ATR-FTIR spectroscopy when combined with machine learning. Tumours as small as 0.2 cm^3^ were correctly identified. The data representing both high-grade and low-grade gliomas is promising for applicability in the clinic. The strong test performance corresponds with our previously published work using this same spectroscopy-based platform technology for the stratification of cancer patients versus controls and also differentiating between specific tumour types [30,32]. The high sensitivities, specificities and balanced accuracies illustrate that the spectroscopic liquid biopsy approach is a reliable diagnostic tool for brain cancer patients with the ability to be implemented for early detection screening on patients that are symptomatic.

The ability to differentiate between glioma and non-glioma patients at such an early stage of cancer development could have a significant impact on patients. Surgery, when tumours are smaller, is more likely to achieve macroscopic gross total resection, which correlates with outcome. Surgical morbidity will also be lower. Complete resection in surgery is not always feasible because of the location of the tumour and involvement of critical neurovascular structures that may compromise the patient’s functional outcome if injured [41]. We previously reported that ATR-FTIR can also help discriminate tumour sub-types [30], so for patients with tumours in eloquent brain regions not suitable for resection, liquid biopsy may obviate the need for, and risk of, surgery.

The spectroscopy-based test reported here could be used as a triage method to fast track and prioritise patients who need medical imaging. Existing brain imaging referral guidelines based on symptoms alone perform poorly and future guidelines should combine symptoms with clinical tests for imaging triage [5].

## 5. Conclusions

Utilising blood serum for ATR-FTIR spectroscopy has been developed for the early detection of brain tumours which can have significant impact on patient treatment plans, surgical outcomes, patient prognosis and quality of life. The stratification between glioma and non-glioma patients have previously been published, as well as classification between specific brain cancer types, both with excellent sensitivity, specificity and balanced accuracies, illustrating a reliable diagnostic tool. The size of brain tumour that is detectable via this technique was explored here for both high-grade and low-grade tumours. A range of tumour sizes, tumour types, patient ages and a gender balance were implemented within the study to represent a greater population. Patients were split into two groups depending on the parameters used for MRI calculations, either T1-weighted with contrast enhancement or T2-weighted/FLAIR. Both groups had sensitivities, specificities and balanced accuracies above 88% with majority around 90% or greater.

While liquid biopsies that use genetic material are emerging as a diagnostic tool, for early detection of cancer it is limited in the very low concentrations of tumour DNA present and for CNS tumours specifically there is the added limitation due to the blood–brain barrier. This technique here offers an alternative approach by detection of immune response to tumours which can be differentiated against healthy patients. Tumours as small as 0.2 cm^3^ were detected within this study providing a screening method and potential diagnostic tool for early detection of brain tumours on patients that present with the non-specific symptoms. This quick, high-throughput technique can reduce the patient diagnostic pathway which will ultimately lead to improved treatments and patient prognosis.

## Figures and Tables

**Table 1:** Summary of sensitivity, specificity and balanced accuracies with each classification for both T1 and T2/FLAIR tumour volumes versus controls. Partial least squares-discriminant analysis (PLS-DA), support vector machine (SVM) and random forest (RF). Top performing model is highlighted in grey.

Cohort	Model	Sensitivity (%)			Specificity (%)			Balanced Accuracy (%)		
		Mean	SD	95% CI	Mean	SD	95% CI	Mean	SD	95% CI
T1	PLS-DA	98.5	3.6	±1.0 97.5–99.5	95.1	3.4	±0.9 94.2–96.0	96.8	2.2	±0.6 96.2–97.4
	SVM	96.2	5.6	±1.5 94.7–97.7	94.2	4.5	±1.2 93.0–95.4	95.2	3.4	±0.9 94.3–96.1
	RF	96.2	6.1	±1.7 94.5–97.9	93.4	5.2	±1.4 92.0–94.8	94.8	3.7	±1.0 93.8–95.8
T2/FLAIR	PLS-DA	88.7	8.6	±2.4 86.3–91.1	94.7	3.9	±1.1 93.6–95.8	91.7	4.6	±1.3 90.4–93.0
	SVM	81.9	10.1	±2.8 79.1–84.7	95.5	3.8	±1.0 94.5–96.5	88.7	5.2	±1.4 87.3–90.1
	RF	82.8	10.7	±2.9 79.9–85.7	95.5	4.4	±1.2 94.3–96.7	89.2	5.6	±1.5 87.7–90.7

## Data Availability

All data is presented within the manuscript or supplementary materials. For any further information contact corresponding authors.

## Supplementary Materials

The following are available online at https://www.mdpi.com/article/10.3390/cancers13153851/s1, Figure S1: Scree plot illustrating the percentage of the total explained variance (TEV) in the first 10 principal components for T1 tumour volumes and controls, Figure S2: Principal component analysis of the first and second dimensions with cancer patients blue and asymptomatic controls yellow. The eclipses in each class represent a 95% confidence interval. Values in parentheses is the TEV in each principal component, Figure S3: Contribution plot illustrating the percentage that the top 20 wavenumbers contribute to the overall separation of the two classes, Figure S4: Pre-processed spectra of the fingerprint region (1800–1000 cm^−1^) for the T1 glioma cohort, with the mean highlighted in red, Figure S5: Scree plot illustrating the percentage TEV in the first 10 principal components for T2/FLAIR tumour volumes and controls, Figure S6: Principal component analysis of the first and second dimensions with cancer patients blue and healthy controls yellow. The eclipses in each class represent a 95% confidence interval. Values in parentheses is the TEV in each principal component, Figure S7: Contribution plot illustrating the percentage that the top 20 wavenumbers contribute to the overall separation of the two classes; Table S1: Summary of patients included within the study, Table S2: Each tumour included in the T1 and T2/FLAIR test sets with prediction results for the first iteration. Tick represents a correct prediction. Partial least squares-discriminant analysis (PLS-DA), support vector machine (SVM) and random forest (RF).

## References

[1] Brown T.J., Brennan M.C., Li M., Church E.W., Brandmeir N.J., Rakszawski K.L., Patel A.S., Rizk E.B., Suki D., Sawaya R. (2016). Association of the extent of resection with survival in glioblastoma. JAMA Oncol..

[2] Ozawa M., Brennan P.M., Zienius K., Kurian K.M., Hollingworth W., Weller D., Grant R., Hamilton W., Ben-Shlomo Y. (2019). The usefulness of symptoms alone or combined for general practitioners in considering the diagnosis of a brain tumour: A case-control study using the clinical practice research database (CPRD) (2000–2014). BMJ Open.

[3] Swann R., McPhail S., Witt J., Shand B., Abel G.A., Hiom S., Rashbass J., Lyratzopoulos G., Rubin G. (2018). Diagnosing cancer in primary care: Results from the National Cancer Diagnosis Audit. Br. J. Gen. Pract..

[4] Ozawa M., Brennan P.M., Zienius K., Kurian K.M., Hollingworth W., Weller D., Hamilton W., Grant R., Ben-Shlomo Y. (2018). Symptoms in primary care with time to diagnosis of brain tumours. Fam. Pract..

[5] Zienius K., Chak-Lam I., Park J., Ozawa M., Hamilton W., Weller D., Summers D., Porteous L., Mohiuddin S., Keeney E. (2019). Direct access CT for suspicion of brain tumour: An analysis of referral pathways in a population-based patient group. BMC Fam. Pract..

[6] Villanueva-Meyer J.E., Mabray M.C., Cha S. (2017). Current Clinical Brain Tumor Imaging. Neurosurgery.

[7] Jin L., Min L., Jianxin W., Fangxiang W., Tianming L., Yi P. (2014). A survey of MRI-based brain tumor segmentation methods. Tsinghua Sci. Technol..

[8] Farace P., Giri M.G., Meliadò G., Amelio D., Widesott L., Ricciardi G.K., Dall’Oglio S., Rizzotti A., Sbarbati A., Beltramello A. (2011). Clinical target volume delineation in glioblastomas: Pre-operative versus post-operative/pre-radiotherapy MRI. Br. J. Radiol..

[9] Angulakshmi M., Lakshmi Priya G.G. (2017). Automated brain tumour segmentation techniques—A review. Int. J. Imaging Syst. Technol..

[10] Gray E., Butler H.J., Board R., Brennan P.M., Chalmers A.J., Dawson T., Goodden J., Hamilton W., Hegarty M.G., James A. (2018). Health economic evaluation of a serum-based blood test for brain tumour diagnosis: Exploration of two clinical scenarios. BMJ Open.

[11] Gray E., Cameron J.M., Butler H.J., Jenkinson M.D., Hegarty M.G., Palmer D.S., Brennan P.M., Baker M.J. (2021). Early economic evaluation to guide the development of a spectroscopic liquid biopsy for the detection of brain cancer. Int. J. Technol. Assess. Health Care.

[12] Vogt S., Löffler K., Dinkelacker A.G., Bader B., Autenrieth I.B., Peter S., Liese J. (2019). Fourier-transform infrared (FTIR) spectroscopy for typing of clinical *Enterobacter cloacae* complex isolates. Front. Microbiol..

[13] Gajjar K., Trevisan J., Owens G., Keating P.J., Wood N.J., Stringfellow H.F., Martin-Hirsch P.L., Martin F.L. (2013). Fourier-transform infrared spectroscopy coupled with a classification machine for the analysis of blood plasma or serum: A novel diagnostic approach for ovarian cancer. Analyst.

[14] Ollesch J., Drees S.L., Heise H.M., Behrens T., Brüning T., Gerwert K. (2013). FTIR spectroscopy of biofluids revisited: An automated approach to spectral biomarker identification. Analyst.

[15] Sala A., Anderson D.J., Brennan P.M., Butler H.J., Cameron J.M., Jenkinson M.D., Rinaldi C., Theakstone A.G., Baker M.J. (2020). Biofluid diagnostics by FTIR spectroscopy: A platform technology for cancer detection. Cancer Lett..

[16] Ollesch J., Heinze M., Heise H.M., Behrens T., Brüning T., Gerwert K. (2014). It’s in your blood: Spectral biomarker candidates for urinary bladder cancer from automated FTIR spectroscopy. J. Biophotonics.

[17] Ellis D.I., Goodacre R. (2006). Metabolic fingerprinting in disease diagnosis: Biomedical applications of infrared and Raman spectroscopy. Analyst.

[18] Mattox A.K., Bettegowda C., Zhou S., Papadopoulos N., Kinzler K.W., Vogelstein B. (2019). Applications of liquid biopsies for cancer. Sci. Transl. Med..

[19] Wan J.C.M., Massie C., Garcia-Corbacho J., Mouliere F., Brenton J.D., Caldas C., Pacey S., Baird R., Rosenfeld N. (2017). Liquid biopsies come of age: Towards implementation of circulating tumour DNA. Nat. Rev. Cancer.

[20] El Messaoudi S., Rolet F., Mouliere F., Thierry A.R. (2013). Circulating cell free DNA: Preanalytical considerations. Clin. Chim. Acta.

[21] Breitbach S., Tug S., Simon P. (2012). Circulating cell-free DNA. Sports Med..

[22] Mouliere F., El Messaoudi S., Pang D., Dritschilo A., Thierry A.R. (2014). Multi-marker analysis of circulating cell-free DNA toward personalized medicine for colorectal cancer. Mol. Oncol..

[23] Mouliere F., Robert B., Arnau Peyrotte E., Del Rio M., Ychou M., Molina F., Gongora C., Thierry A.R. (2011). High fragmentation characterizes tumour-derived circulating DNA. PLoS ONE.

[24] Fiala C., Diamandis E.P. (2018). Utility of circulating tumor DNA in cancer diagnostics with emphasis on early detection. BMC Med..

[25] Cohen J.D., Li L., Wang Y., Thoburn C., Afsari B., Danilova L., Douville C., Javed A.A., Wong F., Mattox A. (2018). Detection and localization of surgically resectable cancers with a multi-analyte blood test. Science.

[26] Upadhyay N., Waldman A.D. (2011). Conventional MRI evaluation of gliomas. Br. J. Radiol..

[27] Smith B.R., Baker M.J., Palmer D.S. (2018). PRFFECT: A versatile tool for spectroscopists. Chemom. Intell. Lab. Syst..

[28] Butler H.J., Smith B.R., Fritzsch R., Radhakrishnan P., Palmer D.S., Baker M.J. (2018). Optimised spectral pre-processing for discrimination of biofluids via ATR-FTIR spectroscopy. Analyst.

[29] Butler H.J., Ashton L., Bird B., Cinque G., Curtis K., Dorney J., Esmonde-White K., Fullwood N.J., Gardner B., Martin-Hirsch P.L. (2016). Using Raman spectroscopy to characterize biological materials. Nat. Protoc..

[30] Cameron J.M., Butler H.J., Smith B.R., Hegarty M.G., Jenkinson M.D., Syed K., Brennan P.M., Ashton K., Dawson T., Palmer D.S. (2019). Developing infrared spectroscopic detection for stratifying brain tumour patients: Glioblastoma multiforme vs. lymphoma. Analyst.

[31] Baker M.J., Trevisan J., Bassan P., Bhargava R., Butler H.J., Dorling K.M., Fielden P.R., Fogarty S.W., Fullwood N.J., Heys K.A. (2014). Using Fourier transform IR spectroscopy to analyze biological materials. Nat. Protoc..

[32] Butler H.J., Brennan P.M., Cameron J.M., Finlayson D., Hegarty M.G., Jenkinson M.D., Palmer D.S., Smith B.R., Baker M.J. (2019). Development of high-throughput ATR-FTIR technology for rapid triage of brain cancer. Nat. Commun..

[33] Smith B.R., Ashton K.M., Brodbelt A., Dawson T., Jenkinson M.D., Hunt N.T., Palmer D.S., Baker M.J. (2016). Combining random forest and 2D correlation analysis to identify serum spectral signatures for neuro-oncology. Analyst.

[34] Ali J., Khan R., Ahmad N., Maqsood I. (2012). Random forests and decision trees. Int. J. Comput. Sci. Issues.

[35] Palmer D.S., O’Boyle N.M., Glen R.C., Mitchell J.B.O. (2007). Random forest models to predict aqueous solubility. J. Chem. Inf. Model..

[36] Otto M. (2016). Pattern recognition and classification. Chemometrics.

[37] Theakstone A.G., Rinaldi C., Butler H.J., Cameron J.M., Confield L.R., Rutherford S.H., Sala A., Sangamnerkar S., Baker M.J. (2021). Fourier-transform infrared spectroscopy of biofluids: A practical approach. Transl. Biophotonics.

[38] Gromski P.S., Muhamadali H., Ellis D.I., Xu Y., Correa E., Turner M.L., Goodacre R. (2015). A tutorial review: Metabolomics and partial least squares-discriminant analysis—A marriage of convenience or a shotgun wedding. Anal. Chim. Acta.

[39] Hajian-Tilaki K. (2013). Receiver Operating Characteristic (ROC) curve analysis for medical diagnostic test evaluation. Casp. J. Intern. Med..

[40] Efron B., Tibshirani R.J. (1994). An Introduction to the Bootstrap.

[41] Wang L., Liang B., Li Y.I., Liu X., Huang J., Li Y.M. (2019). What is the advance of extent of resection in glioblastoma surgical treatment—A systematic review. Chin. Neurosurg. J..

