# Supplementary material for: Rapid Spectroscopic Liquid Biopsy for the Universal Detection of Brain Tumours
Source: Cancers (Basel). 2021 Jul 30;13(15):3851. doi: 10.3390/cancers13153851 (PMC8345395; doi:10.3390/cancers13153851)
Supplement: Supplementary file 1 [file cancers-13-03851-s001.zip › cancers-1292359-supplementary.pdf]

cancers

MDPI

Supplementary Materials: Rapid Spectroscopic Liquid Biopsy for the Universal Detection of Brain Tumours

Methods

Patient selection

Table S1. Summary of patients included within the study.

	GBM	AA	A	OD	OA	Controls	Total
Total no.	47	13	12	15	3	87	177
Age range (mean)	43–79 (60.9)	30–58 (47.6)	30–82 (54.7)	33–69 (41.1)	63–71 (67.0)	20–69 (34.4)	20–82 (45.2)
Gender F/M	23/24	5/8	7/5	8/7	1/2	48/39	92/85
Size of tumour (cm^3)	0.2–96.9	6.9–156.0	2.8–226.2	8.4–111.7	5.6–86.7	N/A	0.2–226.2

Sensitivity, specificity and balanced accuracy

Sensitivity and specificity are based on the number of correct and incorrect predictions in the test set. The sensitivity corresponds to the correct identification of patients with a glioma and the specificity correlates to the identification of patients without a glioma (asymptomatic controls).

$$Sensitivity = \frac{TP}{TP + FN} = \frac{TP}{P}$$

$$Specificity = \frac{TN}{TN + FP} = \frac{TN}{N}$$

When a patient with a glioma has 5 or more spectra (out of 9 repeats) correctly identified we record this as a true positive (TP). Patients without glioma who have 5 or more correct identifications reports as a true negative (TN). False positives (FP) occur when a patient without the disease is incorrectly identified as having a glioma 5 or more times, and a false negative (FN) is when a glioma patient has incorrect identification 5 or more times. P is the number of real positives and N is the number of real negatives.

The overall performance of a binary model can be measured by calculating the balanced accuracy, which is defined as the averaged sensitivity and specificity.

$$Balanced\ accuracy = \left(\frac{TP}{P} + \frac{TN}{N}\right)/2$$

Results

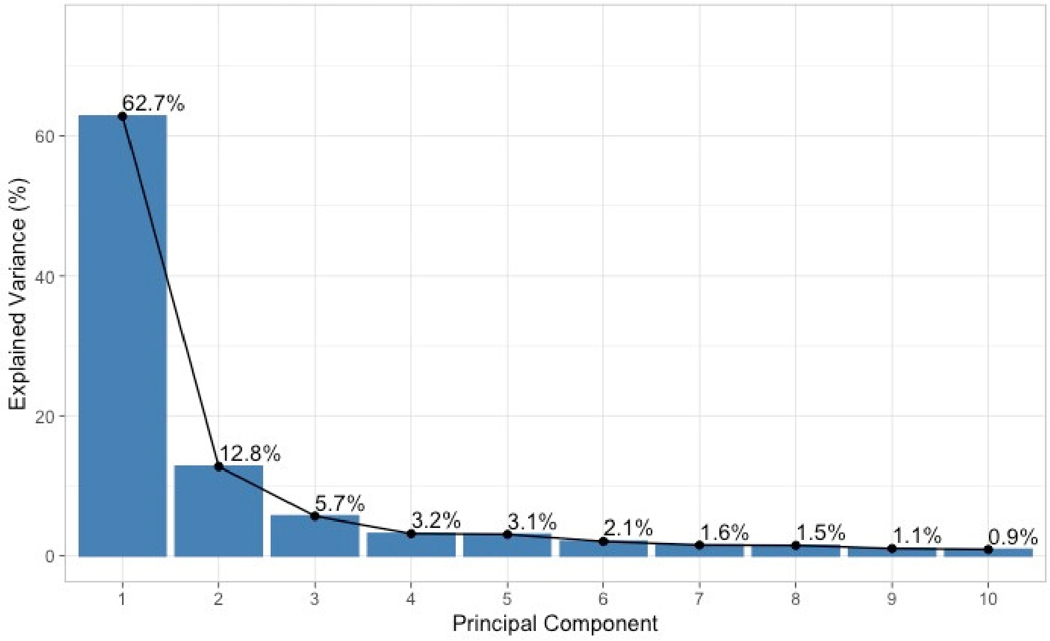

Figure S1. Scree plot illustrating the percentage of the total explained variance (TEV) in the first 10 principal components for T1 tumour volumes and controls.

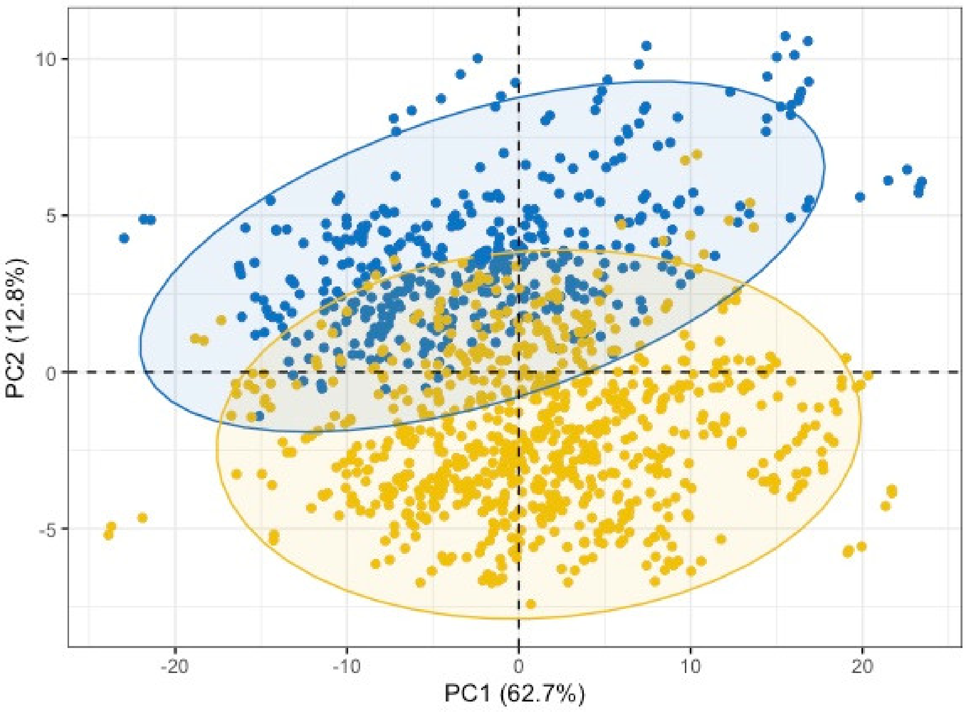

Figure S2. Principal component analysis of the first and second dimensions with cancer patients blue and asymptomatic controls yellow. The eclipses in each class represent a 95% confidence interval. Values in parentheses is the TEV in each principal component.

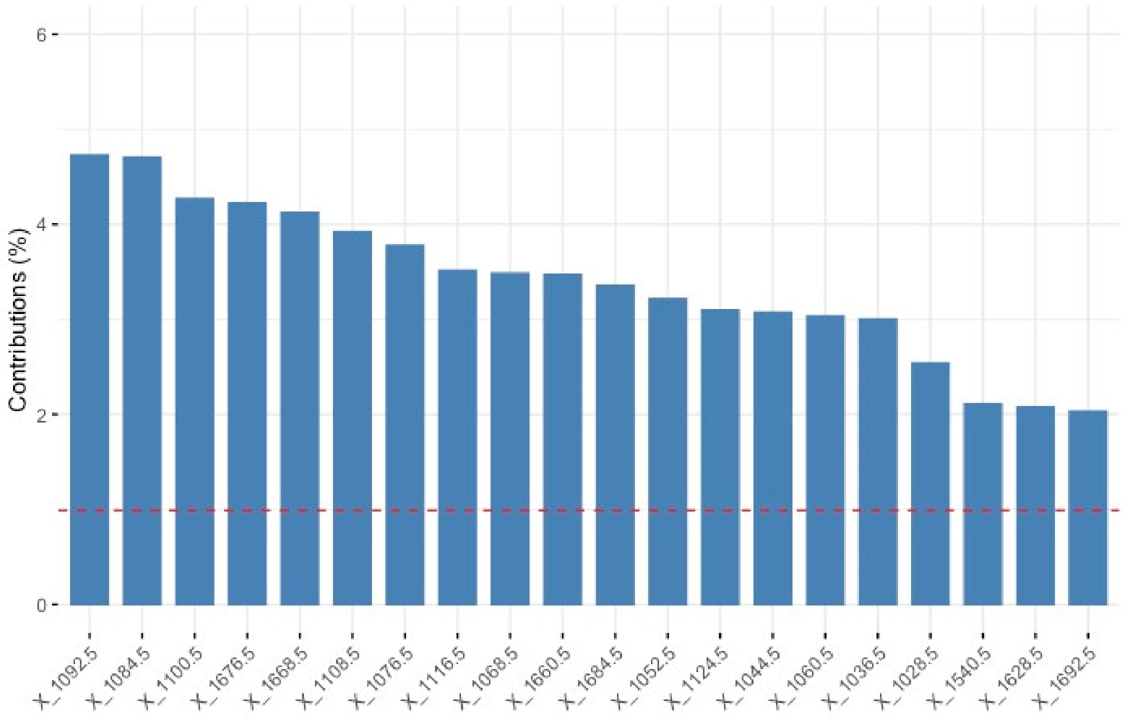

Figure S3. Contribution plot illustrating the percentage that the top 20 wavenumbers contribute to the overall separation of the two classes.

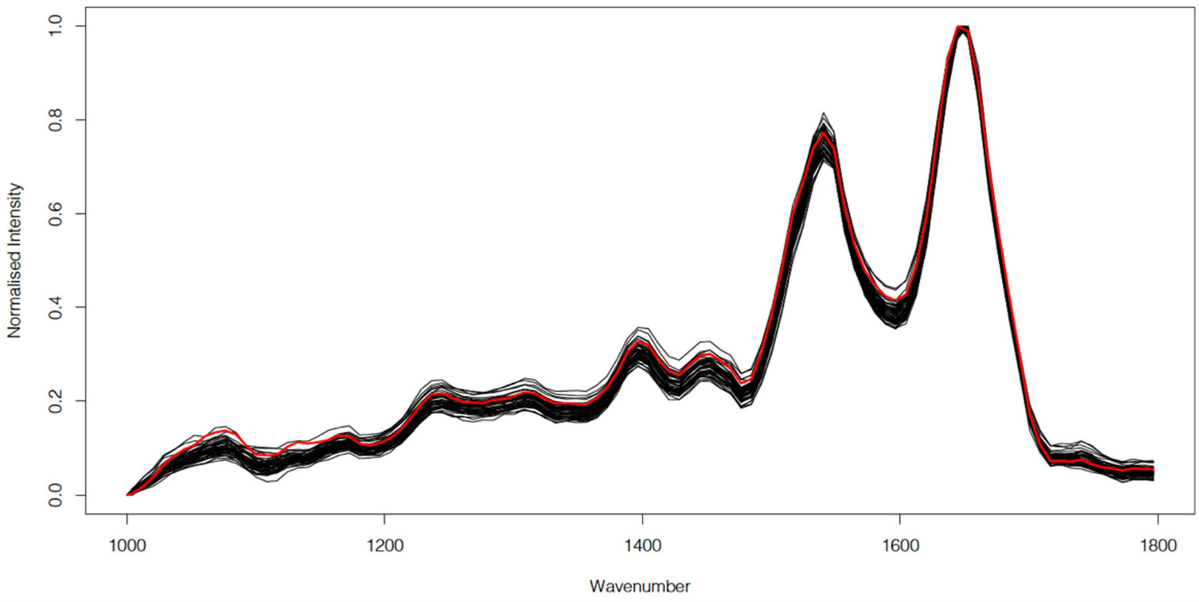

Figure S4. Pre-processed spectra of the fingerprint region (1800–1000 cm^{-1}) for the T1 glioma cohort, with the mean highlighted in red.

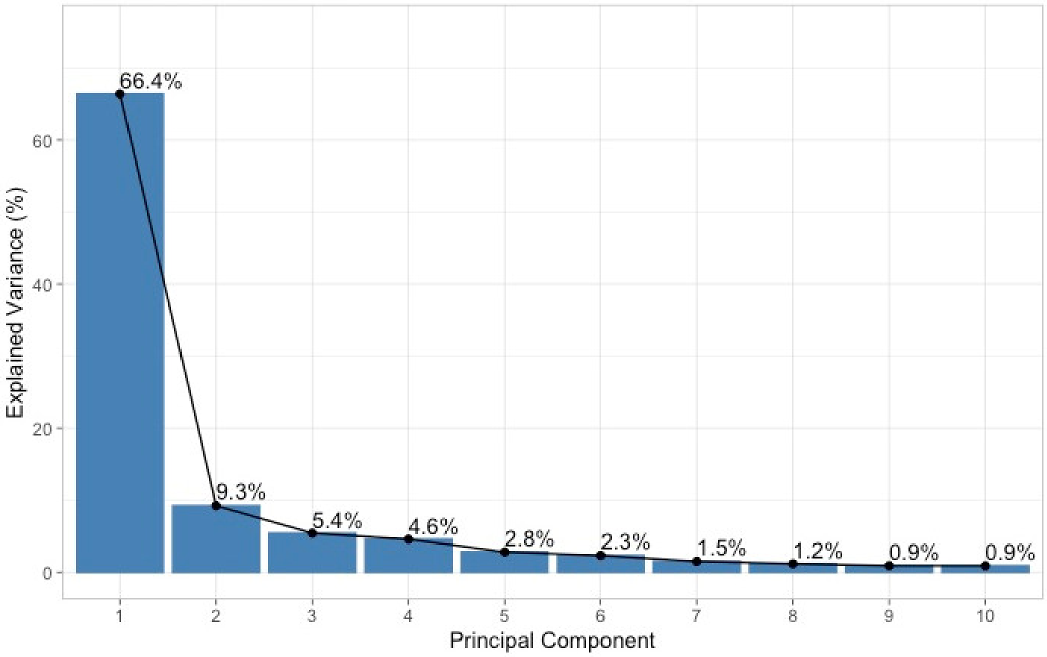

Figure S5. Scree plot illustrating the percentage TEV in the first 10 principal components for T2/FLAIR tumour volumes and controls.

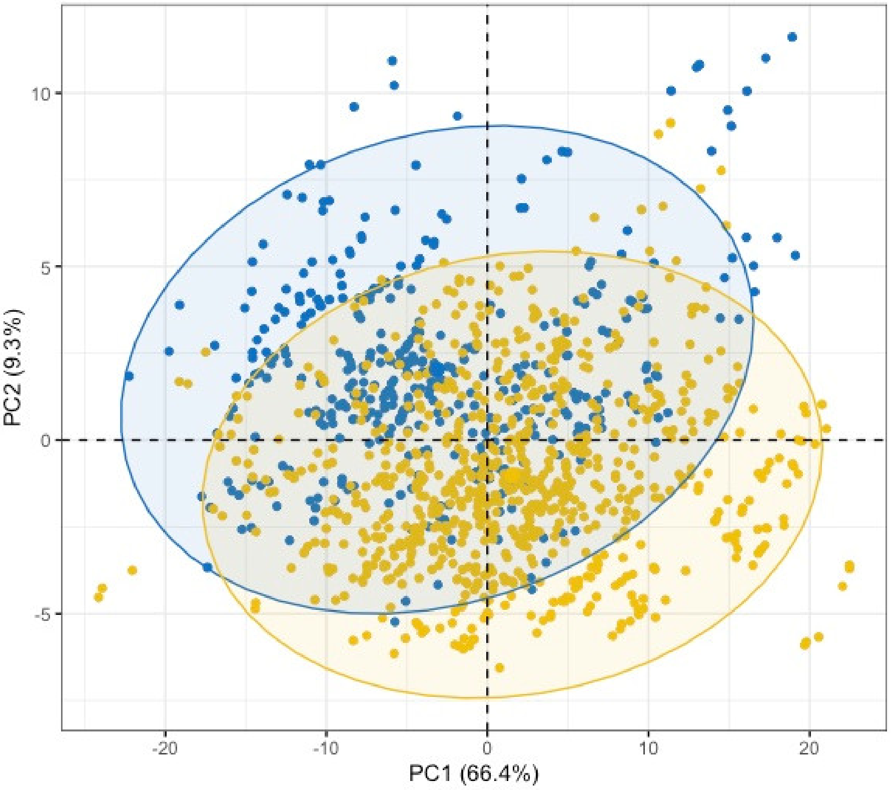

Figure S6. Principal component analysis of the first and second dimensions with cancer patients blue and healthy controls yellow. The eclipses in each class represent a 95% confidence interval. Values in parentheses is the TEV in each principal component.

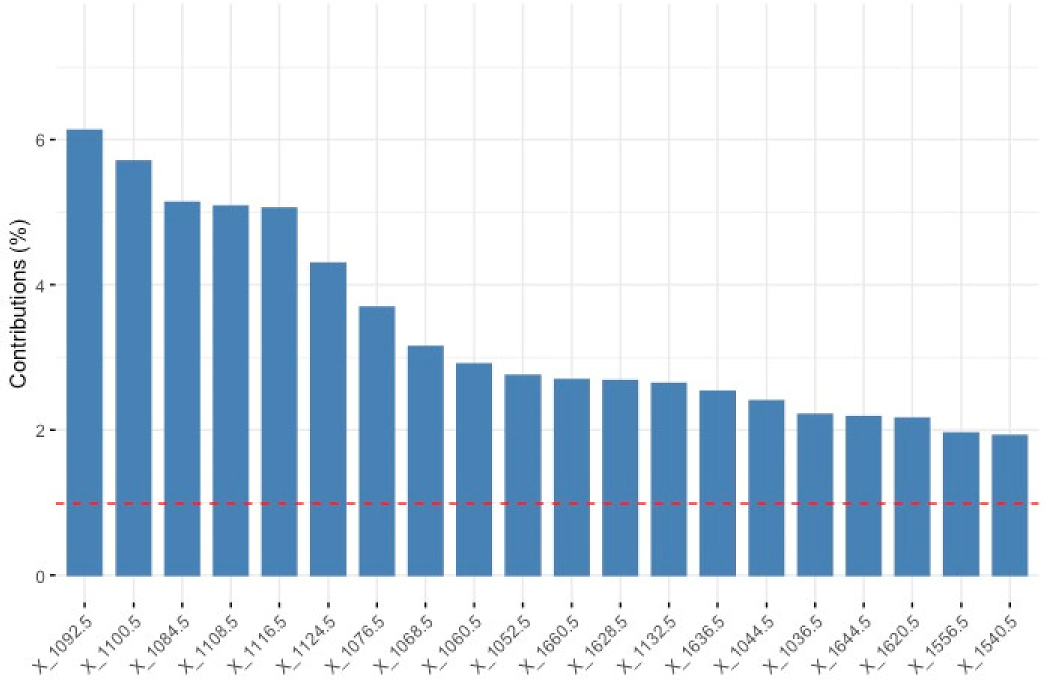

Figure S7. Contribution plot illustrating the percentage that the top 20 wavenumbers contribute to the overall separation of the two classes.

Table S2. Each tumour included in the T1 and T2/FLAIR test sets with prediction results for the first iteration. Tick represents a correct prediction. Partial least squares-discriminant analysis (PLS-DA), support vector machine (SVM) and random forest (RF).

T1					T2/FLAIR				
Tumour Type	Volume (cm^3)	PLS-DA	SVM	RF	Tumour Type	Volume (cm^3)	PLS-DA	SVM	RF
GBM	0.2	✓	✓	✓	OA	5.6	✓	✓	✗
GBM	3.8	✓	✓	✓	OD	8.4	✓	✓	✓
GBM	7.8	✓	✓	✓	OD	13.6	✓	✓	✓
GBM	12	✓	✓	✓	AA	14.4	✓	✓	✓
GBM	23.1	✓	✓	✓	OD	15	✓	✓	✓
GBM	32.7	✓	✓	✓	A	23.3	✗	✗	✓
GBM	35.1	✓	✓	✓	A	27.4	✓	✓	✓
GBM	38.6	✓	✓	✓	OD	38.7	✓	✗	✓
GBM	40	✓	✓	✓	OD	51.1	✓	✓	✓
GBM	40.3	✓	✓	✓	AA	78.4	✓	✓	✓
GBM	43	✓	✓	✓	A	89.1	✓	✓	✓
GBM	45.5	✓	✓	✓	AA	132.2	✓	✓	✓
GBM	52	✓	✓	✓					
GBM	52.6	✓	✓	✓					
